# Programmed Cell Death Protein 1 (PD-1) and Programmed Cell Death Ligand 1 (PD-L1) Immunotherapy: A Promising Breakthrough in Cancer Therapeutics

**DOI:** 10.7759/cureus.44582

**Published:** 2023-09-02

**Authors:** Abdelrahman Abaza, Faten Sid Idris, Humna Anis Shaikh, Ilma Vahora, Kiran Prasad Moparthi, Majdah T Al Rushaidi, Meghana Reddy Muddam, Omobolanle A Obajeun, Arturo P Jaramillo, Safeera Khan

**Affiliations:** 1 Pathology, California Institute of Behavioral Neurosciences & Psychology, Fairfield, USA; 2 Pediatrics, California Institute of Behavioral Neurosciences & Psychology, Fairfield, USA; 3 General Surgery, Saint George's University School of Medicine, Chicago, USA; 4 College of Medicine, Sri Venkata Sai (SVS) Medical College, Mahabubnagar, IND; 5 General Practice, California Institute of Behavioral Neurosciences & Psychology, Fairfield, USA; 6 Psychology, California Institute of Behavioral Neurosciences & Psychology, Fairfield, USA; 7 Paediatrics, California Institute of Behavioral Neurosciences & Psychology, Fairfield, USA; 8 General Practice, Universidad Estatal de Guayaquil, Machala, ECU; 9 Internal Medicine, California Institute of Behavioral Neurosciences & Psychology, Fairfield, USA

**Keywords:** clinical trials, immune check-point inhibitor, cancer therapeutics, immunotherapy, pathology

## Abstract

The advent of immune checkpoint inhibitors has revolutionized cancer therapy by leveraging the body's immune system to combat malignancies effectively. Among these groundbreaking agents, programmed cell death protein 1 (PD-1) and programmed cell death ligand 1 (PD-L1) inhibitors have emerged as pivotal therapeutic approaches. PD-L1, a key protein expressed on the surface of various cells, including cancer cells, plays a central role in immune regulation by interacting with the programmed cell death protein 1 (PD-1) receptor on T-cells leading to immune suppression. The substantial increase in PD-L1 expression on cancer cell surfaces has driven the exploration of PD-1/PD-L1 inhibitors as potential immunotherapeutic agents. These inhibitors are monoclonal antibodies designed to impede the PD-L1 and PD-1 interaction and disrupt the immunosuppressive signal, thereby reinvigorating the anti-tumor immune response mediated by activated T-cells. Clinical trials investigating PD-1/PD-L1 inhibitors have demonstrated remarkable efficacy in the treatment of diverse advanced or metastatic cancers, including leukemia, non-small cell lung (NSCLC), hepatocellular, melanoma, gastric, colorectal, and breast cancers, among others. Regulatory approvals have been granted for both monotherapy and combination therapy with other cancer treatments, encompassing chemotherapy and additional immune checkpoint inhibitors. While PD-1/PD-L1 inhibitors have exhibited significant success, they are not devoid of challenges. The emergence of intrinsic or acquired resistance, as well as immune-related adverse events, warrants thorough investigation and management. Consequently, researchers have embarked on combination trials to augment the therapeutic potential of PD-1/PD-L1 inhibitors and surmount resistance mechanisms.

## Introduction and background

Cancer continues to present a formidable global health challenge, necessitating continuous efforts to advance therapeutic strategies and improve patient outcomes. For over a century, researchers have recognized the potential of the immune system to target tumor cells, inspiring the development of cancer immunotherapy. A significant breakthrough in this field emerged with the utilization of antibodies to block T-cell immune regulatory checkpoints, revealing remarkable curative effects [1]. T lymphocytes play a pivotal role in tumor targeting. Notably, the inhibition of cytotoxic T lymphocyte-associated antigen 4 (CTLA-4) became the first successful checkpoint blockade in cancer immunotherapy, highlighting the critical role of costimulatory signaling in T-cell activation [2].

At the forefront of immune checkpoint regulators stands programmed cell death ligand 1 (PD-L1), a critical protein expressed in various cell types, including tumor cells and antigen-presenting cells (APCs). PD-L1 plays a pivotal role in immune regulation through its interaction with the programmed cell death protein 1 (PD-1) receptor on T-cells, leading to the suppression of the immune response and facilitating tumor immune evasion and uncontrolled tumor growth. Notably, researchers have unveiled increased PD-L1 expression on the surfaces of cancer cells, offering a plausible mechanism through which tumors circumvent immune surveillance [3].

This article endeavors to present a comprehensive review of the PD-1/PD-L1 immune checkpoint, elucidating its underlying mechanisms and delving into the clinical applications of PD-1/PDL-1 inhibitors. Drawing upon a selection of key studies and clinical trials, we aim to provide an in-depth assessment of the current state of knowledge surrounding PD-1/PDL-1 inhibition as an exciting breakthrough in cancer therapy. By examining the clinical outcomes of PD-1/PDL-1 inhibitors, we aspire to shed light on the potential of this innovative immunotherapeutic approach to enhance patient outcomes and facilitate the exploration of novel combination therapies.

## Review

Methods

Adopting the Preferred Reporting Items for Systematic Reviews and Meta-Analyses (PRISMA) framework facilitated the execution of a comprehensive and transparent systematic review.

Search Strategy

To ensure the inclusion of relevant and high-quality data, the research team employed an extensive search strategy across various reputable research resources, including PubMed, PubMed Central, Nature, and Springer. The selection of appropriate keywords was a crucial component of our search process, aimed at capturing pertinent literature related to our research topic. The keywords selected were "Cancer Immunotherapy," "Programmed Cell Death Ligand 1," "Programmed Cell Death Protein 1," "Immune Checkpoint Inhibitors," "Pembrolizumab Clinical Trials," and "Immunotherapy Resistance."

Inclusion and Exclusion Criteria

Table 1 presents a clear representation of the criteria used for inclusion and exclusion in the analysis.

**Table 1:** Inclusion and exclusion criteria employed throughout the meticulous selection process of articles

Inclusion criteria	Exclusion criteria
1. Articles written in English	1. Articles written in different languages
2. Articles released within the last 20 years	2. Articles released before 20 years
3. Papers with relevance to the topic discussed	3. Articles without relevance to the topic discussed
4. Systematic reviews, review articles, and randomized clinical trials	4. Case studies, observational studies, and grey articles

Analysis of Study Quality

In this review, four authors independently conducted thorough evaluations of pertinent literature to identify reliable information. Initially, the abstracts of retrieved articles were thoroughly scrutinized to assess their alignment with the predefined inclusion criteria. Subsequently, those articles matching the inclusion criteria underwent a comprehensive full-text assessment of the credibility of the ideas presented. Once deemed reliable, selected articles were meticulously read to verify the precision of the ideas they presented. Following a rigorous selection process, nine review articles were chosen based on their adherence to the Scale for the Assessment of Narrative Review Articles (SANRA) checklist [4]. The SANRA checklist utilizes a scoring system with a range from zero to two for each standard. A high-quality review article indicates a score of more than or equal to ten. 

Table 2 presents a detailed account of the quality assessment results for the review articles that met the criteria of the SANRA checklist.

**Table 2:** The evaluations using the SANRA checklist for all the review articles included in this study. Table 2 presents the scores obtained from the SANRA checklist, offering a comprehensive assessment of the quality of each included review article. SANRA, Scale for the Assessment of Narrative Review Articles

Publication	Jiang et al. [5]	Yi et al. [6]	Shen et al. [7]	Sunshine et al. [8]	Cha et al. [9]	Kythreotou et al. [10]	Ren et al. [11]	Wang et al. [12]	Hudson et al. [13]
Article's justification	1	2	2	2	1	2	2	2	2
Expression of specific objectives or articulation of inquiries	2	2	1	2	2	1	2	2	2
Account of the literature exploration	2	1	2	1	2	2	1	2	2
Referencing	2	2	2	1	2	2	2	2	1
Rational scientific deduction	1	2	2	2	1	2	1	2	2
Suitable depiction of information	2	2	2	2	2	2	2	2	2
Score	10	11	11	10	10	11	10	12	11

In addition to the review articles, four clinical trials were selected to further demonstrate the actual efficacy of immune checkpoint inhibitors in the treatment of different types of cancers. 

Table 3 illustrates the evaluation of Cochrane risk-of-bias, which screens for different research biases for the chosen clinical trials.

**Table 3:** Cochrane risk-of-bias for assessment of the selected clinical trials Table 3 presents the analysis of Cochrane risk-of-bias evaluation for the designated clinical trials.

Clinical Trial	Selection bias	Reporting bias	Performance bias	Detection bias	Attrition bias
Gao et al. [14]	Low risk	Intermediate risk	Low risk	Low risk	Low risk
Shitara et al. [15]	Low risk	Low risk	Low risk	Intermediate risk	Low risk
Larkin et al. [16]	Intermediate risk	Low risk	Low risk	Low risk	Low risk
Nanda et al. [17]	Low risk	Low risk	Intermediate risk	Low risk	Intermediate risk

Data Extraction

The utilization of the Preferred Reporting Items for Systematic Reviews and Meta-Analyses (PRISMA) guidelines was of paramount importance in ensuring a high-quality data extraction process. The selection of review articles from various databases was conducted through a meticulous approach, characterized by a stringent methodology that encompassed thorough quality assessments. This approach was employed to discern and eliminate articles lacking relevance or manifesting deficiencies.

**Figure 1:**
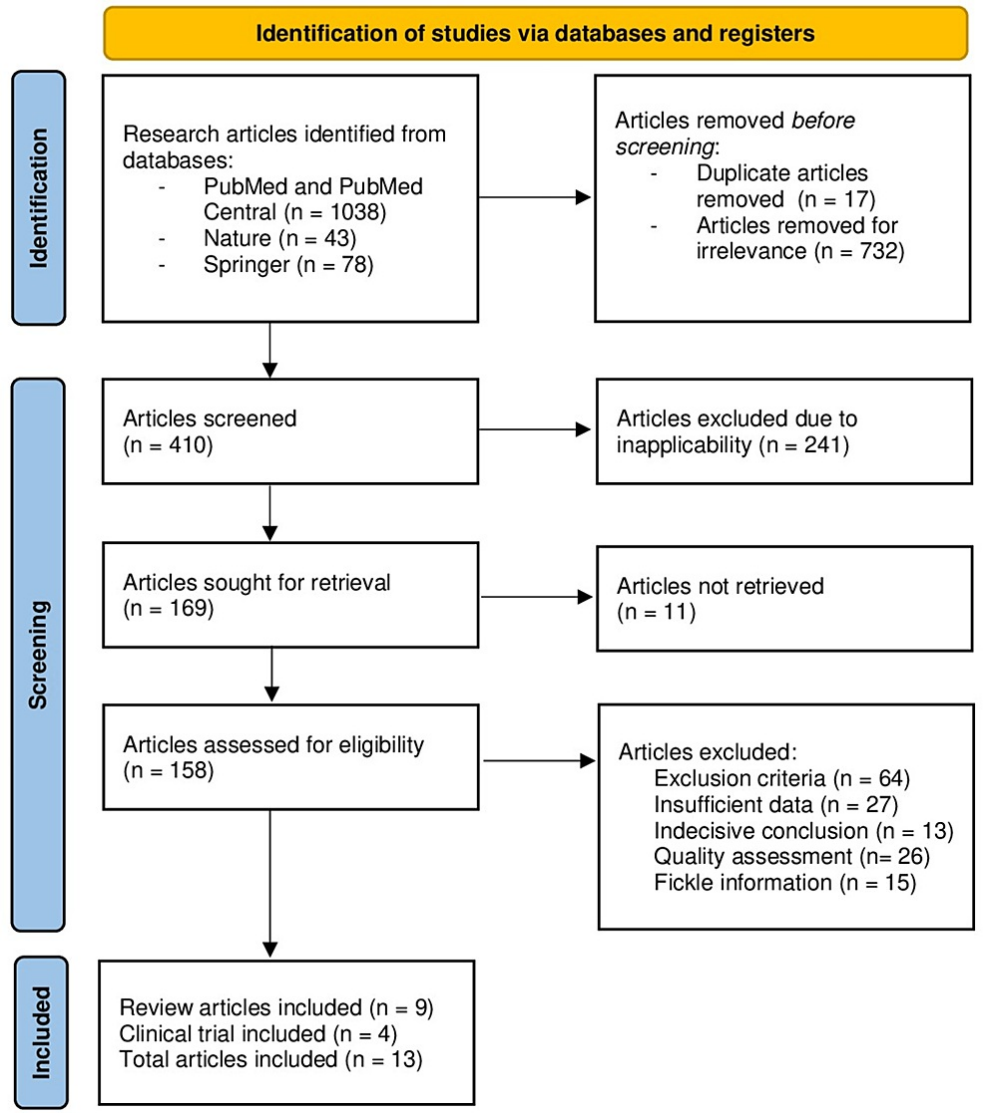
PRISMA flowchart for data extraction Figure 1 provides a visual representation of the data extraction process following the flowchart guidelines outlined by the PRISMA PRISMA, Preferred Reporting Items for Systematic Reviews and Meta-Analyses

Figure 1 illustrates the data extraction procedure using the PRISMA flowchart methodology.

Results

In the initial stage of database exploration, a total of 1159 research articles underwent assessment. Among these, 17 duplicate articles were identified and excluded, and then, an additional 732 articles were excluded due to their lack of relevance. Subsequently, a meticulous review was conducted to gauge the precision of the ideas presented, resulting in the exclusion of 241 research articles due to inapplicability. Through this process, 169 research articles were retained for further eligibility assessment and subjected to multiple screening criteria. Ultimately, 13 research articles emerged as meeting the stringent standards and were deemed worthy of inclusion in our comprehensive review.

Discussion

Over a century ago, scientific explorations revealed the immune system's potential to target malignant cells within the body. As our comprehension of immunology expanded, it became evident that T-cells possess the capability to combat tumor cells, leveraging their distinctive attributes of specificity, memory, and adaptation [18]. Moreover, a groundbreaking avenue in immunotherapy emerged with the advent of antibody-based interventions targeting T-cell immune checkpoints, yielding transformative curative outcomes. The activation of T-cells hinges upon a dual-signal framework necessitating both antigen receptor signaling and cluster of differentiation (CD) 28 costimulatory signaling. A pioneering instance of checkpoint protein inhibition materialized through antibody-mediated blockade of CTLA-4, demonstrating efficacy in cancer immunotherapy. CTLA-4's capacity to relocate to the T-cell surface and engage in competitive binding with CD28, thus impeding the interaction with CD80 and CD86 expressed by APCs, culminates in the dampening of T-cell proliferation and activation. Parallel to this, other T-cell-intrinsic checkpoints such as PD-1 orchestrate inhibitory signals that modulate T-cell function [19,20]. In contemporary times, a surge of scientific exploration has been directed toward unraveling the intricacies of the PD-1/PD-L1 pathway, spurred by its exceptional clinical efficacy, enduring responses, and minimal toxicity. The objective underlying cancer immunotherapies targeting the PD-1/PD-L1 signaling nexus transcends mere enhancement of immune cell function within the tumor microenvironment; rather, it aims to restore immune system equilibrium [21]. 

Structure of PD-1 and PD-L1

PD-L1 is a constituent of the B7 protein family, which represents the ligand of PD-1. Within their architecture, PD-L1 is categorized as a type I glycoprotein featuring IgV and IgC structural domains, alongside a hydrophobic transmembrane segment and a cytoplasmic tail structure domain. Positioned on chromosome nine, the genes encoding this ligand exhibit a remarkable degree of sequence conservation. The dynamic interplay between PD-1 and PD-L1 unfolds predominantly within the intricate confines of the tumor microenvironment. In essence, PD-1, found abundantly on activated T-cells, engages with its ligand counterpart PD-L1, present on select tumor cells and APCs. This engagement initiates a cascade of events, notably culminating in the phosphorylation of tyrosine residues within the PD-1 cytoplasmic region. This phosphorylation event orchestrates the recruitment of Src homology 2 domain-containing protein tyrosine phosphatase-2 (SHP-2). Consequent to this interaction, downstream elements, including spleen tyrosine kinase (Syk) and phospholipid inositol-3-kinase (PI3K), undergo phosphorylation. This series of events collectively leads to the suppression of downstream signaling pathways, with consequential impacts on T-cell biological functions. Crucially, the repercussions encompass compromised lymphocyte proliferation, subdued cytokine secretion, and diminished CTL cytotoxicity. The ramifications of this intricate interplay extend further, inducing apoptosis among tumor-specific T-cells. These adaptive alterations empower tumor cells to elude vigilant immune surveillance by T-cells, thus contributing to immune evasion strategies within the context of tumorigenesis [22].

PD-1 and PD-L1 Functions

The PD-1/PD-L1 pathway assumes a pivotal role across a spectrum of domains encompassing autoimmune diseases, viral infections, and tumor immunity. In physiological equilibrium, this very pathway upholds peripheral immune tolerance, delivering a constructive influence by curbing excessive tissue inflammation and mitigating the emergence of autoimmune disorders. Nonetheless, when tumors emerge and advance, the convergence of PD-1 and PD-L1 casts a suppressive shadow upon the host's anti-tumor immunity. This inhibition paves the way for the evasion of immune surveillance by tumor cells, a multifaceted phenomenon involving: Impairing lymphocyte activation and prompting their apoptosis, curtailing the production of CTL granzymes and perforin, dampening the release of inflammatory cytokines while fostering the secretion of the immune-inhibitory cytokine Interleukin-10, and halting the progression of the T-cell cycle [23,24].

PD-1/PD-L1 Expression in Immune Tolerance and Cancer

The PD-1/PD-L1 axis stands as a pivotal orchestrator of inherent immune equilibrium, wielding influence across diverse tiers of immune tolerance. Following antigen recognition, the emergence of PD-1 on the surface of T-cells plays an important role in shaping the magnitude of the initial T-cell response. The activation of T-cell activity primarily hinges upon a dual signaling mechanism. The main signal is reproduced through the fusion of major histocompatibility complex (MHC)-bound antigens with the T-cell receptor (TCR). The secondary signal is a composite of co-stimulatory and co-inhibitory cues. Notably, the interaction between PD-1 on T-cells and PD-L1 on tumor cells or antigen-presenting cells (APCs) exerts a potent dampening effect on T-cell activation. This interaction can even culminate in T-cell apoptosis, reducing cytokine output, and immunosuppression. The disruption of the PD-1-PD-L1 axis, by contrast, imparts a boost to immune responses directed at invading pathogens [25]. Within the microenvironment of tumor-infiltrating lymphocytes, PD-1 assumes a persistent ascendancy, concomitant with an exploitative elevation of PD-L1 expression by malignant entities. This elevation of PD-1 emerges as a tactical maneuver, enabling malignant cells to deflect immune obliteration. Notably, this dynamic is magnified within contexts characterized by an unceasing presence of antigen-bearing target cells-typified by the landscape of cancer. Within this setting, T-lymphocytes undergo a gradual loss of their effector functions, transitioning toward a debilitated state, aptly designated as dysfunction. Of particular significance, the amplification of PD-1 emerges as the hallmark of these dysfunctional T-cells. Eclipsing the contours of its functional relevance, PD-1 persists as a sentinel in the perpetuation of the dysfunctional state within cancer-stricken tissues. This potent role undertaken by PD-1 significantly contributes to the web of mechanisms harnessed by cancer to elude the vigilant surveillance of the immune system [26].

PD-1/PD-L1 Immune Checkpoint Inhibitors

Having grasped the knowledge that PD-1 signaling puts the brakes on T-cell activities like activation and cytokine release, and recognizing the significant role of PD-1/PD-L1 in cancer's strategy to suppress T-cell activation, scientists and researchers became deeply intrigued. Their focus shifted to crafting specialized antibodies, widely known as monoclonal antibodies, capable of disrupting the PD-1/PD-L1 interaction to counteract T-lymphocytes' suppression and bolster the immune response against cancer cells [27]. Antibodies aimed at PD-1 or its counterpart, PD-L1, breathe new life into the T-cells in a dysfunctional state, rekindling their ability to fend off cancer cells. The resounding achievements observed in clinical trials have paved the way for the Food and Drug Administration (FDA) approval of ten PD-1 monoclonal antibodies (including nivolumab, pembrolizumab, camrelizumab, sintilimab, cemiplimab, prolgolimab, tislelizumab, dostarlimab, toripalimab, and zimberelimab) and three PD-L1 monoclonal antibodies (namely avelumab, durvalumab, and atezolizumab) to combat various forms of cancer [28].

*PD-1/PD-L1 * Inhibitors Implications in Common Human Cancers

Lung cancer: the landscape of lung cancer treatment has been profoundly reshaped by tumor immunotherapy directed at PD-1/PD-L1. Notably, the effectiveness of PD-L1 inhibitors surpasses that of chemotherapy, particularly in advanced non-small cell lung cancer (NSCLC) patients exhibiting elevated PD-L1 levels. This potency is equally evident among patients with previously untreated metastatic squamous NSCLC. Moreover, when considering patients with NSCLC who have undergone prior treatment, a decreased rate of disease progression is more frequently observed in response to PD-1/PD-L1 inhibitors, as opposed to conventional chemotherapy. This observation holds true, particularly for patients with an extensive metastatic burden and an adverse prognosis. In current clinical therapeutics, a strategic alliance between PD-1/PD-L1 immune checkpoint inhibitors and chemotherapeutic agents has emerged as a cornerstone of treatment. This approach attests to the heightened value these inhibitors bring to the therapeutic arsenal. The rapid evolution of anti-PD-1/PD-L1 inhibitors for advanced NSCLC stands as an instrumental factor in enhancing patient outcomes, charting a promising trajectory toward improved prognosis [29,30]. In a recent study, neoadjuvant PD-1 inhibitor sintilimab was administered to individuals with NSCLC. The outcomes revealed that a notable 40.5% of participants achieved a major pathological response, while a commendable 10.8% realized a complete remission at the pathological level [14].

Prostate cancer: currently, PD-1/PD-L1 immune checkpoint inhibitors have ushered substantial clinical advantages for individuals with prostate cancer. A recent study has put forth the notion that synergizing PD-1/PD-L1 checkpoint inhibitors with radiotherapy presents a promising avenue in the management of prostate cancer [31]. However, it is noteworthy that the impact of PD-L1/PD-1 blockade in the context of prostate cancer appears comparatively muted in contrast to its influence on other cancer types. This discrepancy stems from the diminished immunogenicity characterizing prostate cancers [32].

Breast cancer: metastatic triple-negative breast cancer exhibits a prospective reactivity toward immunotherapy involving PD-1/PD-L1 inhibitors. An ongoing study has unveiled an encouraging prospect, delineating the efficacy of PD-1/PD-L1 immune checkpoint inhibitors with chemotherapy as a novel and auspicious clinical trajectory for the treatment of triple-negative breast cancer [33]. In a recent study, the inclusion of PD-1 immune checkpoint inhibitor pembrolizumab alongside conventional neoadjuvant chemotherapy resulted in a doubling of the estimated pathological complete response compared to chemotherapy alone. This effect was particularly pronounced in cases of triple-negative breast cancer [17]. Nonetheless, the application of PD-1/PD-L1 inhibitors in the breast cancer realm remains relatively infrequent. This circumstance is based on the fact that breast cancer tends to manifest a comparatively decreased immunogenicity compared to other tumor types [34].

Gastric cancer: empirical clinical trials have solidified the efficacy of PD-1 targeted therapy as a viable option for individuals with metastatic gastric cancer. The utilization of the anti-PD-1 antibody pembrolizumab has emerged as a beacon of hope, boasting anti-tumor effectiveness and a tolerable safety profile within the realm of treating patients with recurrent or metastatic gastric cancer [35]. Furthermore, the employment of nivolumab and chemotherapy has persistently showcased an improvement in overall survival compared to chemotherapy alone, particularly among patients exhibiting PD-L1 expression [15].

Colorectal cancer: novel therapeutic avenues for colorectal cancer have emerged through the recognition of immune checkpoint inhibitors. These inhibitors have demonstrated evident therapeutic efficacy in cases of metastatic colorectal cancer characterized by deficient mismatch repair or exhibiting high microsatellite instability [36]. Nevertheless, individuals harboring proficient mismatch repair or microsatellite stable tumors have not experienced substantial gains from immunotherapy interventions [37].

Hepatocellular carcinoma: recent clinical trials have unveiled compelling evidence of the notable enhancement in overall survival among patients with hepatocellular carcinoma (HCC) through the implementation of immune checkpoint inhibitor therapy. Exemplary instances include the favorable outcomes observed with PD-1 inhibitors, notably pembrolizumab and nivolumab, showcasing both effectiveness and tolerability in advanced HCC cases [38]. Of particular significance, the synergistic integration of anti-PD-1/PD-L1 antibodies with other therapeutic modalities has emerged as a robust and efficacious strategy in the comprehensive management of HCC [39].

Skin cancers: PD-1 and PD-L1 inhibitors have demonstrated remarkable efficacy in addressing the challenges posed by metastatic skin cancers. Notably, few PD-1 and PD-L1 inhibitors have recently earned approval from the U.S. FDA for the treatment of metastatic cutaneous squamous cell carcinoma and basal cell carcinoma [40]. Furthermore, within the realm of metastatic melanoma, previously untreated patients experienced a substantial improvement in progression-free survival when subjected to the combined intervention of nivolumab and the CTLA-4 checkpoint inhibitor ipilimumab when contrasted with ipilimumab monotherapy [16].

Leukemia: Anti-PD-1/PD-L1 therapy has emerged as a novel and promising immunotherapeutic approach for addressing acute myeloid leukemia (AML). The results of one clinical trial have showcased encouraging rates of response and overall survival among individuals afflicted with refractory AML who underwent treatment with nivolumab and azacitidine. This combination therapy has demonstrated its safety and efficacy, underscoring its potential as a viable and valuable avenue for the treatment of AML [41]. 

PD-1/PD-L1 Inhibitors Resistance Mechanisms

In recent times, the landscape of tumor immunotherapy has been profoundly reshaped by the advent of immune checkpoint blockade therapy targeting the PD-1/PD-L1 axis, leading to a paradigm shift marked by remarkable therapeutic advancements across a diverse spectrum of malignancies. Nonetheless, the emergence of resistance to PD-1/PD-L1 inhibitors in a substantial proportion of patients has cast a formidable shadow over the otherwise promising horizon of this therapeutic approach. This phenomenon not only poses a significant clinical conundrum but also underscores an imperative for a comprehensive understanding of the molecular mechanisms underpinning immune checkpoint inhibitor resistance. Resistance to PD-1/PD-L1 inhibitors manifests in two distinct forms: primary resistance and acquired resistance. Primary resistance encompasses patients who exhibit no clinical response or stable disease upon initiation of PD-1/PD-L1 blockade. This resistance phenotype is attributed to an array of factors, including decreased cancer immunogenicity, inadequate interferon response, alterations in epidermal growth factor receptor (EGFR), exclusion of T-lymphocytes as well as immunosuppression elements within the cancer's microenvironment. Acquired resistance to PD-1/PD-L1 inhibitors entails a scenario wherein these inhibitors initially engender a sustained therapeutic response upon treatment initiation. However, over time, the efficacy of these inhibitors substantially wanes or ceases to evoke a response in a subset of patients. The mechanisms underpinning this phenomenon are linked to factors such as T-lymphocyte exhaustion and functional decline, compromised antigen presentation, acquired genetic mutations, diminished memory lymphocytes, and heightened activation of alternative immune checkpoints such as CTLA-4. Elucidating these complex pathways is pivotal for strategies to decrease the challenge of acquired resistance to PD-1/PD-L1 inhibitors [42].

## Conclusions

Upon careful consideration of the aforementioned details, a resounding conclusion emerges: the PD-1/PD-L1 immune checkpoint inhibitors stand as a cornerstone of great importance in cancer management across a spectrum of diverse malignancies. Their consistent efficacy underscores their pivotal role in shaping modern oncologic and pathologic therapeutics. This overarching impact serves as a testament to the potential that these inhibitors harbor in cancer therapeutics, offering renewed hope and tangible advancements for patients with a myriad of cancer types. As research continues to unfold and unveil further insights, the trajectory of cancer treatment is poised for continued evolution, where PD-1/PD-L1 inhibitors remain at the forefront of innovative strategies aimed at ameliorating the prognosis and quality of life for affected individuals. Although PD-1/PDL-1 inhibitors have demonstrated notable achievements, they are not exempt from encountering obstacles. The emergence of inherent or acquired resistance necessitates in-depth exploration and effective mitigation strategies. As a result, researchers have initiated comprehensive combination therapeutic trials aimed at amplifying the therapeutic efficacy of PD-1/PDL-1 inhibitors and circumventing the mechanisms of resistance.
